# Supplementary material for: Effects of Task Interference on Kinematics and Dual-Task Cost of Running in Early Childhood
Source: Sensors (Basel). 2024 Feb 27;24(5):1534. doi: 10.3390/s24051534 (PMC11154332; doi:10.3390/s24051534)
Supplement: Supplementary file 1 [file sensors-24-01534-s001.zip › sensors-2865442-supplementary.pdf]

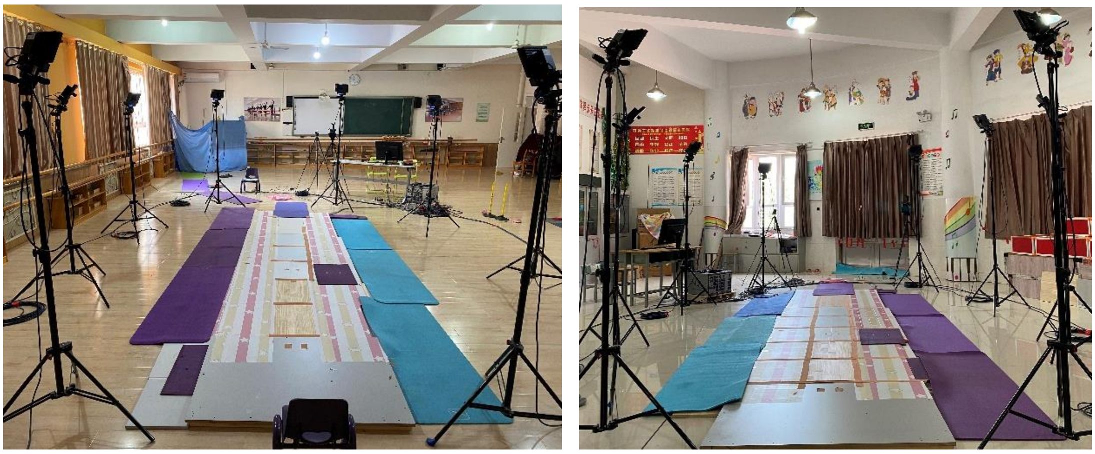

Figure S1 Testing sites in kindergarten and primary school

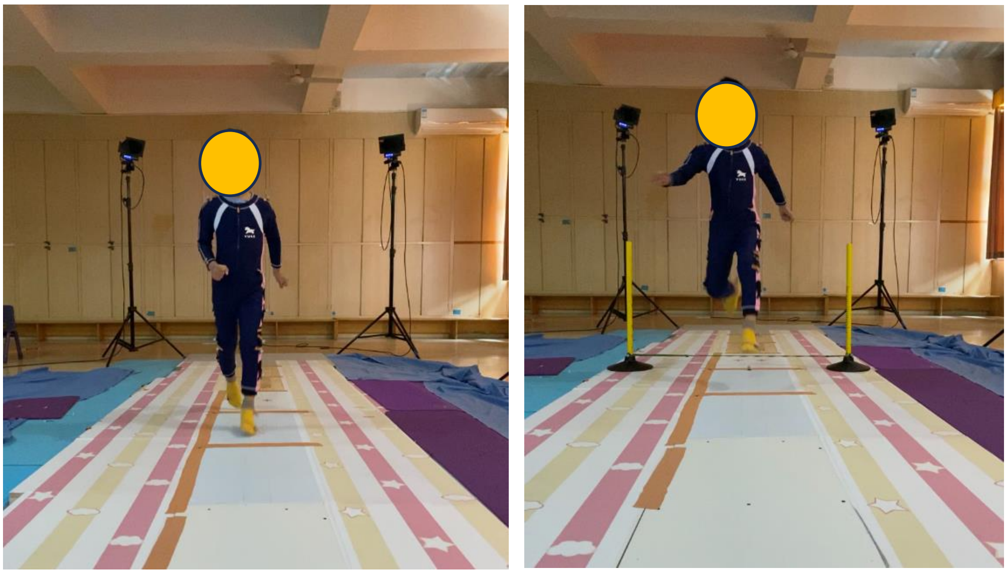

Figure S2 Cognitive dual tasks and obstacle crossing tasks in kindergarten

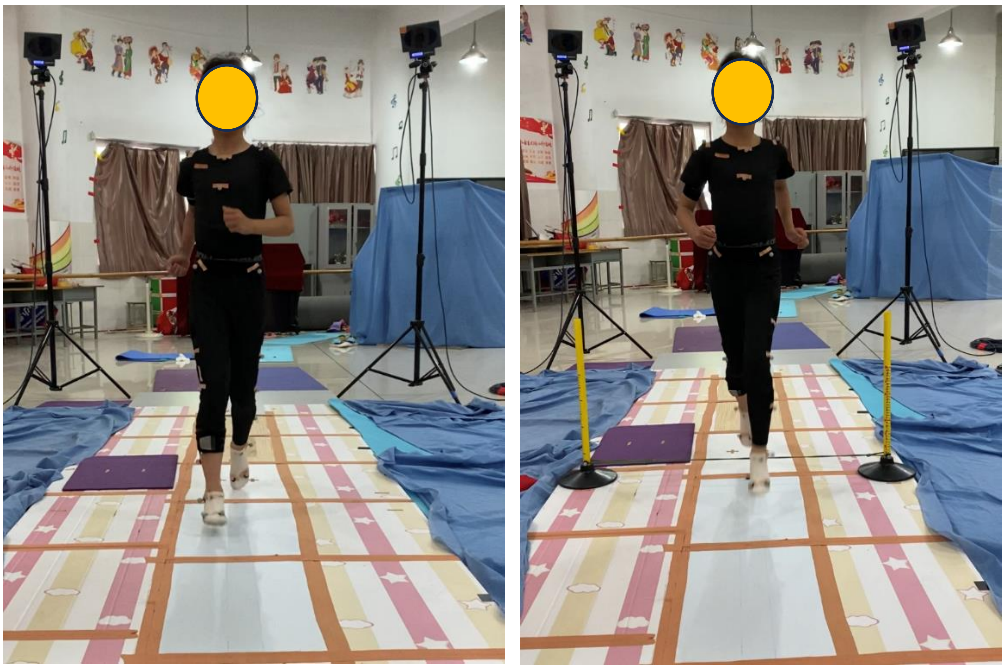

Figure 3 Cognitive dual tasks and obstacle crossing tasks in primary school

Table S1 Main and interaction effect of running spatiotemporal parameters

Dependent variable	Main/interaction effect	*df*	*F*	*sig*	η_p^2
Running cycle time (s)	Task	2	53.775	0.000	0.234
	Age	5	9.343	0.000	0.210
	Task*Age	10	1.601	0.104	0.044
Stance phase (%)	Task	2	71.103	0.000	0.288
	Age	5	2.276	0.050	0.061
	Task*Age	10	1.877	0.052	0.051
Swing phase (%)	Task	2	51.186	0.000	0.225
	Age	5	3.921	0.002	0.100
	Task*Age	10	2.041	0.075	0.055
Stride length (%/height)	Task	2	25.768	0.000	0.131
	Age	5	3.864	0.002	0.102
	Task*Age	10	1.597	0.106	0.045
Step length (m)	Task	2	42.639	0.000	0.203
	Age	5	22.015	0.000	0.397
	Task*Age	10	1.645	0.093	0.047
Stride width (m)	Task	2	2.253	0.107	0.013
	Age	5	0.436	0.823	0.012
	Task*Age	10	1.690	0.081	0.046
Cadence (step/min)	Task	2	82.754	0.000	0.320
	Age	5	9.166	0.000	0.207
	Task*Age	10	1.760	0.067	0.048
Speed (%/height/s)	Task	2	104.388	0.000	0.372
	Age	5	2.864	0.016	0.075
	Task*Age	10	2.184	0.058	0.058

Table S2 Main and interaction effect of joint angles

Dependent variable	Main/interaction effect	*df*	*F*	*sig*	η_p^2
Hip ROM (deg)	Task	2	238.584	0.000	0.578
	Age	5	3.508	0.005	0.092
	Task*Age	10	1.768	0.065	0.048
Knee ROM (deg)	Task	2	612.148	0.000	0.779
	Age	5	2.593	0.027	0.069
	Task*Age	10	1.528	0.128	0.042
Ankle ROM (deg)	Task	2	115.539	0.000	0.399
	Age	5	3.799	0.003	0.098
	Task*Age	10	1.878	0.053	0.051
Hip Landing Angle (deg)	Task	2	79.976	0.000	0.315
	Age	5	2.423	0.037	0.065

	Task*Age	10	1.262	0.251	0.035
Hip Pushing Angle (deg)	Task	2	11.987	0.000	0.064
	Age	5	3.241	0.008	0.085
	Task*Age	10	1.574	0.113	0.043
Knee Landing Angle (deg)	Task	2	83.777	0.000	0.325
	Age	5	4.600	0.001	0.117
	Task*Age	10	1.838	0.053	0.05
Knee Pushing Angle (deg)	Task	2	19.958	0.000	0.103
	Age	5	0.629	0.678	0.018
	Task*Age	10	1.478	0.146	0.041
Ankle Landing Angle (deg)	Task	2	37.743	0.000	0.178
	Age	5	10.131	0.000	0.225
	Task*Age	10	4.150	0.000	0.107
Ankle Pushing Angle (deg)	Task	2	0.546	0.580	0.003
	Age	5	5.132	0.000	0.129
	Task*Age	10	1.118	0.347	0.031
Hip angular velocity	Task	2	140.671	0.000	0.447
	Age	5	5.812	0.000	0.143
	Task*Age	10	0.496	0.893	0.014
Knee angular velocity	Task	2	192.372	0.000	0.525
	Age	5	16.968	0.000	0.328
	Task*Age	10	0.730	0.696	0.021
Ankle angular velocity	Task	2	99.375	0.000	0.364
	Age	5	5.950	0.000	0.146
	Task*Age	10	1.186	0.299	0.033

Table S3 Main and interaction effect of difference task costs

Dependent variable	Main/Interaction effect	*df*	*F*	*sig*	η_p^2
Speed (%/height/s)	Task	1	43.155	**0**	0.197
	Age	5	3.847	**0.002**	0.099
	Task*Age	5	0.4	0.848	0.011
Stride length (%height)	Task	1	10.754	**0.001**	0.058
	Age	5	3.609	**0.004**	0.093
	Task*Age	5	0.445	0.817	0.012
